# Daily Walking and Life Expectancy of Elderly People in the Iowa 65+ Rural Health Study

**DOI:** 10.3389/fpubh.2013.00011

**Published:** 2013-04-18

**Authors:** Hani M. Samawi

**Affiliations:** ^1^Jiann-Ping Hsu College of Public Health, Georgia Southern UniversityStatesboro, GA, USA

**Keywords:** life expectancy, daily walk, hazard ratio, health conditions, proportional hazards model, Cox regression, Kaplan Meier estimate, survival function

## Abstract

The purpose of this paper is to investigate the hypothesis that outdoor daily walking, as an exercise, has an effect on the rate of mortality among those elderly people in the Iowa 65+ Rural Health Study (RHS). RHS is a prospective longitudinal cohort study of 8 years follow-up from 1981 to 1989. It consists of a random sample of 3,673 individuals (1,420 men and 2,253 women) aged 65 or older living in Washington and Iowa counties of the State of Iowa. Our analysis was conducted only on those non-institutional individuals who could without any help walk across a small room; this reduced the total number of individuals in the study to 2,717. Moreover, a total of 923 individuals died during the period of the study. The life histories of those individuals were obtained and divided into two cohorts; one containing 1,134 who exercise daily by walking and the other containing 1,583 who do not exercise daily by walking. The interviewers asked participants about 17 medical conditions, from which 13 are included in our study. We found that daily walking exercise is related inversely to total mortality before and after adjusting for the other factors in particular for age group and health conditions. We observed that hazard ratio (HR) of death was the highest among those individuals having a history of cancer (HR = 2.971) and history of stroke (HR = 2.127). However, individuals with a history of stroke in the “daily walking group” have HR = 0.856 and their risk of death were reduced by 81% compared to those in no “daily walking group.”

## Introduction

Since the time of the ancient Greeks, physical exercise has been praised as an adjunct to good health (1). Studies during the past 20 years have confirmed the value of physical activity in relationship to life expectancy. Physiological and psychological benefits of regular exercise in case of athletes, sedentary people, and coronary heart disease patients are investigated by many researchers, for example see (1, 2, 3). Moreover, walking as one of the physical activities has been studied, revealing an inverse relationship with coronary heart disease, stroke, diabetes, and other health problems, see for example (4, 5, 6, 7, 8, 9, 10, 11, 12).

The importance of walking to cardiovascular health is being appreciated, yet there is a long standing debate about whether exercises also extends life expectancy; see for example: (13, 14, 15, 16) Paffenbarger et al. (17) studied 16,936 Harvard alumni, aged 35–74, in 1986 and reported that a small gradient effect of walking led to a 21 percent lower risk of death as distance was increased from less than three miles to nine or more miles per week. Their report adds new evidence to support the claim that adequate walking exercise preserve life and desirable quality of living into old age.

MacRae et al. (18) examined the effect of 12-week and 22-week walking program endurance capacity, physical activity level, mobility, and quality of life in ambulatory nursing home residents identified as having low physical activity levels and low walk endurance capacities.

They found that, after 12 weeks of walking, members of the experimental group significantly improved their maximal walk endurance time by 77% and their distance by 92%, without any significant change in walk speed. They recommended that future research use walking plus strength-training program to examine the effects of a combined exercise program on health outcomes.

In this paper we investigate the relationship between outdoor walking as a daily exercise and time to death hazard from any cause in the Iowa 65+ Rural Health Study (RHS). The hypothesis that daily walking exercise has an impact on life expectancy is examined in these 8 years of follow-up study of two cohorts of daily walking and no daily walking while controlling the effects of some health conditions and other related factors by using the proportional hazards (PHs) model as discussed below.

## Materials and Methods

Rural Health Study is a prospective longitudinal cohort study of 3,673 individuals (1,420 men and 2,253 women) aged 65 or older living in Washington and Iowa counties of Iowa. This study is one of four studies supported by the National Institute on Aging and collectively referred to as Established Populations for Epidemiological Studies of the Elderly (EPESE), see (19).

The life histories of 2,717 non-institutional individuals who could walk across a small room without any help were obtained from RHS and divided into two cohorts; one containing 1,134 who exercised daily by walking during the 8 years of follow-up and the other containing 1,583 who did not exercise daily by walking during the same period. In the RHS, interviewers asked participants about 17 medical conditions, from which 13 are included in our study (Table 1). The questionnaires used for annual interviews were designed to require the construction of some cumulative variables, including the history of each medical condition, created over the course of successive follow-up studies in addition to follow-up question about daily walking. Because of variation in the wording of the questions during follow-up interviews, different methods have been used to determine whether or not individuals had a history of these conditions at particular time points during the initial 8 years of the investigation as detailed by Rubenstein and Lemke (19).

**Table 1:** **Frequency table and description of the study variables**.

Variable name	Description of the variable	Frequency	Percent
Arches	Trouble with fallen arches or flat feet	0574	21.13
Buncoros	Trouble with bunions, corns, or calluses on his feet	1081	39.79
Dailywk	Do you have daily walking?	1134	41.74
Hist_ANB	History of anemia at the baseline	0300	11.04
Hist_ARB	History of arthritis or rheumatism	1933	71.14
Hist_ASB	History of asthma at baseline	0155	05.70
Hist_BPB	History of high blood pressure at baseline	1167	42.95
Hist_DIB	History of diabetes at baseline	0250	09.20
Hist_EMB	History of emphysema at baseline	0230	08.47
Hist_HAB	History of heart attack at baseline	0345	12.70
Hist_KIB	History of kidneys at baseline	0773	28.45
Hist_PHB	History of phlebitis at baseline	0354	13.03
Hist_PKB	History of Parkinson’s disease at baseline	0024	00.88
Hist_STB	History of stroke at baseline	0146	05.37
Hist_ULB	History of ulcers at baseline	0388	14.28
HistSRCB	Self reported cancer history	0410	15.09
JNTpain	Joints pain at baseline	1777	65.40
JNTstiff	Stiffness in joints at baseline	1388	51.09
Legpain	Legs pain at baseline	1522	56.02
Othfoot	Other foot pains at baseline	0604	22.23
Stroll	Can you walk a half mile?	2285	84.10
Toenails	Trouble with fingernail or toenails	0655	24.11

The baseline interviews were conducted in person during 1981 and 1982. Our study consisted of analyzing data of all non-institutional individuals who could without any help walk across a small room in the RHS. The data base included 2,717 individuals (1,054 men and 1,663 women). Information about the two cohorts of daily walking and no daily walking and other variables in this study were obtained from baseline interviews. Information about time to death from any cause was measured from time of baseline interviews, which is time zero for all individuals, to death or the end of follow-up at 8 years. Status variable (censoring) was measured by the subject being alive at the end of the 8 years period. Because of the non-linear nature of age variable in the under study survival model, age variable has been categorized to six age groups 65–69, 70–74, 75–79, 80–84, 85–89, and 90+. The other variables in our study were categorical as defined in Table 1 below.

### Methods of the analysis

**Figure 1:**
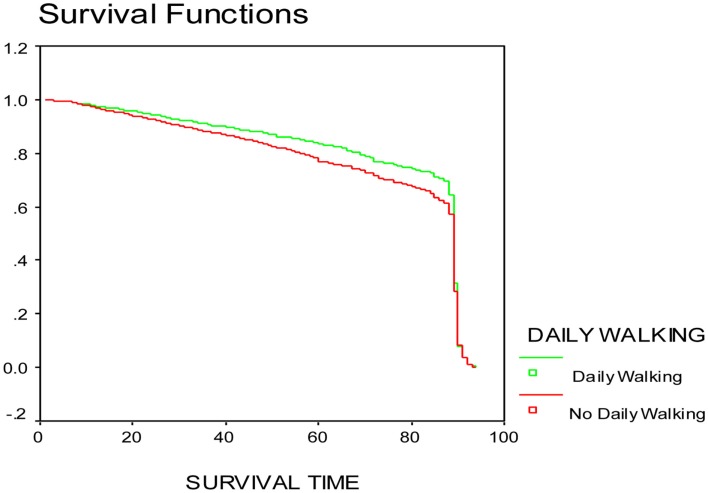
**Kaplan Meier survival function estimate for daily walking variable**.

The main analysis focused on daily walking exercise exposure, with other walking activities, namely stroll, and available health conditions as possible covariates thereof. Initial descriptive survival analysis, Kaplan Meier survival function estimates (20) were obtained to investigate the nature of the relationship between the daily walking exercise and survival time (Figure 1). Figure 1 indicates that the survival functions of the daily walking group and no daily walking group have the same shape, but the one for the daily walking group has higher estimated probability at each point of time than that for the no daily walking group. This indicates that daily walking exercise may be beneficial.

To evaluate the relationship between daily walking and mortality it was necessary to control for several other factors which were likely to be associated with both the mortality rate and the exposure. Controlling of these confounding factors were achieved by adjusting for those factors in the PH model; however to make adjustment for what kind of people are being compared in the various risk set, a stratified analysis over age groups has been used. Significance tests based on stratified Cox score test, Wald test for the univariate analysis, and Likelihood Ratio Test (LRT) for the multivariate Cox regression analysis were used. The effects were then computed as Maximum Likelihood Estimate (MLE) from stratified Cox partial likelihood function.

Model building strategies of selecting a “best” model have been used here to build the PH model; however, since we have 23 possible covariates to select from, we divided the available covariates to two groups, first group consisted of the most significant and biological important covariates, the second group consisted of the rest of the available covariates. The best model selection method is used in this study in both groups of covariates; then the final model has been selected from both groups. The PH assumption for the final model covariates has been tested, concluding that, history of heart attack failed to meet the PH assumption. Therefore, the PH model used to analyze the data is

$$\lambda (t|\underline{x};\;z)=\lambda_{o}(t)\;e^{\beta^{\prime }\;\underline{x}+yz^{*}\;\log(t)},$$

where λ = hazard ratio (HR), (β ’ and γ) is the set of coefficients corresponding to the final model covariates $\underline{x}$ and *z* respectively, *z* = History of heart attack at baseline, log(*t*) = Natural logarithm of survival time, and $\underline{x}$ is the set of covariates other than history of heart attack in the final model (Table 3) Diagnostics such as Δβ and fitted value plots for the final model has been carried out by using EGRET program. The Δβ method showed no significant change in the final model after deleting an outlier (highest Δβ).

## Results

Estimates of each univariate/covariate effect and the results of testing of their significance based on LRT and Score tests as well as estimates for the HRs are listed in Table 2. From the univariate analysis in Table 2, daily walking exercise has a strong negative relationship with total mortality, having a HR of 0.851. Persons with the no daily walking exercises has a 1.2 risk of dying as compared to persons who exercised daily by walking despite if their age group (Age-group variable was not significant). All listed diseases in Table 2 had a positive relationship with total mortality except for Hist_ARB, Arches, Buncorns, JNTpain, JNTftiff, Legpain, and Othfoot. This may be due to these diseases not being life threatening. Strolling was negatively related to total mortality.

**Table 2:** **Stratified univariate analysis for daily walking data using Cox regression model**.

Variables	Coefficient estimates	SE	LRT *P*-value	Score test *P*-value	95% Confidence interval		
					Lower	Hazard	Upper
Buncoros	−0.1881	0.0757	0.012	0.013	0.714	0.829	0.961
Dailywk	−0.1615	0.0755	0.031	0.032	0.676	0.851	0.884
Hist_ARB	−0.2209	0.0780	0.005	0.005	0.688	0.802	0.934
Hist_ASB	0.3456	0.1400	0.019	0.013	1.074	1.413	1.860
Hist_DIB	0.6809	0.1060	<0.001	<0.001	1.606	1.976	2.430
Hist_EMB	0.7688	0.1070	<0.001	<0.001	1.750	2.157	2.658
Hist_HAB	0.6916	0.0899	<0.001	<0.001	1.674	1.997	2.382
Hist_KIB	0.1481	0.0786	0.062	0.059	0.994	1.160	1.353
Hist_STB	0.7133	0.1230	<0.001	<0.001	1.604	2.041	2.597
Hist_ULB	0.1580	0.0998	0.120	0.113	0.963	1.171	1.424
HistSRCB	0.2648	0.0952	0.007	0.005	1.081	1.303	1.570
JNTpain	−0.1122	0.0754	0.137	0.136	0.771	0.894	1.036
JNTstiff	−0.1500	0.0731	0.040	0.040	0.746	0.861	0.993
Sex	−0.7417	0.0733	<0.001	<0.001	0.413	0.476	0.550
Stroll	−0.6444	0.0852	<0.001	<0.001	0.444	0.525	0.620

Multivariate analysis showed some reduction in risk of death in different age groups (Table 3). Some of the factors became non-significant after stratifying for age, and adjusting for daily walking and sex. Insignificant factors were Hist_ANB, Hist_BPB, Hist_PHB, Hist_PKB, Hist_ULB, JNTpain, JNTstiff, Legpain, Othfoot, Toenails, and Arches. Heart attack was strongly positive in relation to total mortality using univariate analysis; however, after adjusting for other factors in the multivariate model interacting with log(*t*), heart attack effect and heart attack interaction with log(*t*) became negatively related to total mortality. This negative relation with total mortality may be due to the benefits of regular exercise in the case of coronary heart disease patients. Daily walking HR was reduced from 0.851 in univariate analysis to 0.745 after adjusting for other factors in the multivariate model. Women age 65+ have less risk of dying compared to men at the same age. The HR for sex variable was 0.476 in univariate analysis and slightly decreased to 0.470 after adjusting for other factors in the multivariate model.

**Table 3:** **Final stratified multivariate analysis for daily walking data using Cox model**.

Covariate	Coefficient	SE	*P*-value	Hazard ratio
Dailywk	−0.2941	0.134	0.028	0.7452
Hist_STB	0.7548	0.157	<0.001	2.1270
Dailywk*Hist_STB	−0.6161	0.264	0.020	0.5401
Stroll	−0.5908	0.110	<0.001	0.5539
Sex	−0.7552	0.080	<0.001	0.4699
Hist_DIB	0.3870	0.136	0.004	1.4730
Hist_HAB	−0.1454	0.175	0.405	0.8647
Hist_EMB	0.4741	0.111	<0.001	1.6070
HistSRCB	1.0890	0.242	<0.001	2.9710
Hist_KIB	0.2627	0.105	0.012	1.3000
Hist_ARB	−0.3890	0.106	<0.001	0.6777
Buncoros	−0.0935	0.100	0.349	1.0988
HistSRCB*Stroll	−0.6502	0.211	0.002	
Dailywk*Hist_ARB	0.3701	0.161	0.022	
Stroll*Hist_HAB	0.4925	0.191	0.010	
Bucorns*Hist_DIB	0.5670	0.220	0.010	
Bucorns*Hist_KIB	−0.3097	0.164	0.059	
HistSRCB*Hist_ARB	−0.4190	0.209	0.045	
Hist_HAB*log(*t*)	−0.3215	0.111	0.004	

Table 4 shows that daily walking is an effect modifier for Hist_STB. Individuals with a history of stroke in the daily walking have a 0.856 HR and reduced their risk of death by 81% compared to those individuals with a history of stroke in the no daily walking group.

**Table 4:** **Hazard ratio of mortality for daily walking by the history of some diseases**.

History of the disease	Daily walking	Hazard ratio for stroke	Hazard ratio for arthritis	Hazard ratio for buncoros
No	No	1.000	1.000	1.000
No	Yes	0.745	0.745	0.745
Yes	No	2.127	0.678	0.910
Yes	Yes	0.856	0.731	0.679

Hist_ARB was negatively related to total mortality with a 0.678 HR of death. Individuals with a Hist_ARB reduced the risk of death by 14.1% in the daily walking group (Table 4). This may be because most of the individuals with Hist-ARB may were women and they are known by their lower risk of death than men.

## Discussion

Survival is the result of a number of interacting influences (many hazardous in character or degree, some beneficial in the same way), including physical activity and other lifestyle elements susceptible to optional adjustment. Their combined effect determines whether increase in life expectancy is feasible or even desirable. The findings reported here represent only a small part of the total picture, but they suggest that daily walking exercise and other walking activity may have a protective effect against any cause of mortality after adjusting for age groups studied. They also indicate that walking may add to the years of life expectancy.

The Iowa 65+ RHS group may not be typical of the general elderly population, because the subjects are primarily from rural areas. The data shows that daily walking implies increased life expectancy for elderly 65+ men and women. It is likely that some individuals in this study increased their exercise and received treatment for some of the listed health conditions (Table 1) during the 8 years of study. Such change would minimize or maximize the importance of walking activities in the present findings.

Table 4 shows the difference in mortality between the daily walking group and the no daily walking group for individuals with a history of stroke, Arthritis, and Buncoros. The trend of lower risk of death in daily walking group compared to no daily walking group may reflect a stage of optimal benefit from daily walking activity for elderly people.

Moreover, Table 4 shows that the effect of daily walking is not independent of the health condition relating to walking as for history of Buncoros, because this condition usually reduces the ability to walk. However, it is not the case when the patient has a history of arthritis. We found that arthritis to be beneficial. This may be due to their ongoing life-style. The data in Table 3 offer evidence of interaction between Buncoros and Hist_KIB and Hist_DIB. Interaction between Buncoros and Hist_KIB had a negative relationship with total mortality. Table 4 surprisingly showed that individuals with a Hist_ARB reduced the risk of death by approximately 14.1% in the no daily walking group. This extra benefit of life expectancy for those having a history of arthritis in the no daily walking group may come from the effect of ongoing health care and special diet.

The data in Tables 2 and 3 do offer some implications as a brief preliminary analysis of survival in various age and daily walking groups. In view of the data base and the consistency represented in the study, it seems likely that the observed patterns are predictive. The present study adds new evidence to support this view.

Further analysis controlling for competing risk factors seems to be a reasonable analysis. On the other hand, extended follow-up of Iowa 65+ subjects may reveal trends of aging or at least show at what stage of life the roles of exercise and other natural therapies have no effect.

## Conflict of Interest Statement

The authors declare that the research was conducted in the absence of any commercial or financial relationships that could be construed as a potential conflict of interest.
